# Supplementary material for: MetaRibo-Seq measures translation in microbiomes
Source: Nat Commun. 2020 Jun 29;11:3268. doi: 10.1038/s41467-020-17081-z (PMC7324362; doi:10.1038/s41467-020-17081-z)
Supplement: Supplementary file 10 — Supplementary Data 7 [file 41467_2020_17081_MOESM10_ESM.zip › File2/Confidence_High_Taxonomy/402373_out.krona.html]

Javascript must be enabled to view this page.

members
magnitude
magnitudeUnassigned
count
unassigned
taxon
rank

402373\_out

61

2
superkingdom
61

61
phylum
1239

909932
class
1

1
order
1843489

1
31977
family

1
genus
39948


SRS013940\_contig\_number\_17050
1
1955814
species

class
186801
60

186802
order
60

186803
family
60

60
genus
841

1
2049040
species

SRS054956\_contig\_number\_13874


SRS011084\_contig\_number\_contig-100\_605.171450SRS013158\_contig\_number\_contig-100\_618.36835SRS013951\_contig\_number\_37144SRS014287\_contig\_number\_5146SRS014412\_contig\_number\_3414SRS014613\_contig\_number\_18391SRS015065\_contig\_number\_34927SRS015190\_contig\_number\_15143SRS015264\_contig\_number\_14261SRS015431\_contig\_number\_76470SRS015578\_contig\_number\_contig-100\_1084.177522SRS015579\_contig\_number\_17210SRS015854\_contig\_number\_16909SRS015890\_contig\_number\_1290SRS015960\_contig\_number\_19280SRS016335\_contig\_number\_16353SRS018623\_contig\_number\_11763SRS018984\_contig\_number\_21617SRS019068\_contig\_number\_38792SRS019267\_contig\_number\_324SRS019397\_contig\_number\_11013SRS021484\_contig\_number\_16740SRS024435\_contig\_number\_39291SRS043411\_contig\_number\_13851SRS050422\_contig\_number\_36073SRS050925\_contig\_number\_33422SRS050941\_contig\_number\_6667SRS053335\_contig\_number\_17517SRS055017\_contig\_number\_20816SRS056273\_contig\_number\_10573SRS058070\_contig\_number\_20862SRS063040\_contig\_number\_37413SRS063518\_contig\_number\_35332SRS075341\_contig\_number\_9006SRS076756\_contig\_number\_21474SRS077454\_contig\_number\_11778SRS077641\_contig\_number\_19377SRS098514\_contig\_number\_contig-100\_1318.5193SRS098881\_contig\_number\_7891SRS1041130\_contig\_number\_4987SRS104485\_contig\_number\_651SRS104975\_contig\_number\_323SRS1055022\_contig\_number\_22463SRS1055034\_contig\_number\_8604SRS142503\_contig\_number\_39050SRS142781\_contig\_number\_contig-100\_884.88012SRS143417\_contig\_number\_14396SRS143780\_contig\_number\_18962SRS144537\_contig\_number\_8327SRS144714\_contig\_number\_21012SRS146812\_contig\_number\_25686SRS147919\_contig\_number\_18159SRS148091\_contig\_number\_9470SRS148159\_contig\_number\_25649SRS149181\_contig\_number\_10311SRS893380\_contig\_number\_contig-100\_14.15
species
166486
56

species
1263104
3

SRS014923\_contig\_number\_38718SRS104084\_contig\_number\_10440SRS149879\_contig\_number\_14594
